# Distribution of serum amyloid A and establishment of reference intervals in healthy adults

**DOI:** 10.1002/jcla.23120

**Published:** 2019-11-13

**Authors:** Qian Liu, Yan Li, Fumeng Yang, Tongdao Xu, Li Yao, Jin Sun, Wei Liang

**Affiliations:** ^1^ Department of Laboratory Medicine The Second People's Hospital of Lianyungang Lianyungang China; ^2^ Department of Endocrinology The Second People's Hospital of Lianyungang Lianyungang China

**Keywords:** acute‐phase reactive protein, reference interval, serum amyloid A

## Abstract

**Background:**

Serum amyloid A (SAA) plays a critical role in acute or chronic and is used in clinical laboratories as an indicator of inflammation. The elevated SAA is closely related to inflammation‐mediated diseases, such as liver diseases, autoimmune diseases, metabolism‐related diseases, amyloidosis, and tumors. However, there is no unified population reference interval for SAA. This study aimed to investigate the distribution of SAA in healthy Chinese adults 20‐79 years of age and to establish its population reference interval.

**Methods:**

A total of 2365 healthy subjects met the requirements of this study. The levels of SAA were detected using an AU5821 automatic biochemical analyzer and its original reagents. According to the recommended methods of CLSI C28‐A3 and WS/T 402‐2012, the population reference interval of SAA was established using the unilateral 95th percentile (P_95_), and the 90% confidence interval of upper limits was calculated.

**Results:**

The distributions of SAA levels were not significantly different between sexes (*P*> .05) and also did not differ by age (*P*> .05). Therefore, the population reference interval for SAA was established as an upper limit of 11.0 mg/L (90% confidence interval: 9.3‐12.3 mg/L) by using the method of latex immunoturbidimetry.

**Conclusions:**

Serum amyloid A is closely related to the occurrence and progression of various diseases. The preliminary establishment of a population reference interval for SAA can fully exert its potential clinical value.

**Abbreviations**
ALB: albumin
ALT: alanine aminotransferase
AST: aspartate aminotransferase
CI: confidence interval
CLSI: Clinical and Laboratory Standards Institute
CNAS: China National Accreditation Service for Conformity Assessment
Cr: creatinine
CRP: C‐reactive protein
GGT: gamma‐glutamyltransferase
GLU: glucose
HDL‐C: high‐density lipoprotein cholesterol
IFCC: International Federation of Clinical Chemistry and Laboratory Medicine
IQR: interquartile range
LDL‐C: low‐density lipoprotein cholesterol
SAA: serum amyloid A
TBIL: total bilirubin
TC: total cholesterol
TG: triglyceride
TP: total protein

## 1 INTRODUCTION

Serum amyloid A (SAA) plays n critical role in acute or chronic and is used in clinical laboratories as an indicator of inflammation.^1^ Although both SAA and C‐reactive protein (CRP) are acute‐phase proteins, the detection of SAA is more conclusive than the detection of CRP in patients with viral infections, severe acute pancreatitis, and rejection reactions to kidney transplants.^2^ The elevated SAA is closely related to inflammation‐mediated diseases, such as liver diseases, autoimmune diseases, metabolism‐related diseases, amyloidosis, and tumors.^3^, ^4^, ^5^, ^6^ Besides, in acute‐phase reactions such as acute inflammation and trauma, the concentration of SAA in the blood can be rapidly increased by approximately 1000‐fold within 5‐6 hours under the stimulation of IL‐1, IL‐6, and TNF‐α.^7^, ^8^ Therefore, SAA has important clinical value in the diagnoses, progression, and prognoses of diseases associated with inflammation.

The reference interval is an important indicator for judging whether the test results are normal. As early as the 1980s, the International Federation of Clinical Chemistry and Laboratory Medicine (IFCC) recommended the establishment of reference intervals for laboratories.^9^ The China National Accreditation Service for Conformity Assessment (CNAS) CNAS:2012, 5.5.2 also clearly stated that the laboratory should specify biological reference intervals or clinically determined values, and even document the basis of this regulation.^10^ However, due to the establishment of the reference interval, many factors such as the inclusion and exclusion criteria of the reference individual, large workload, high economic cost, and other factors are constrained and affected.^11^ Therefore, the establishment and application of reference intervals for laboratory analytes are greatly limited. At present, the population reference interval of SAA is mainly derived from kit instructions. Because the reference interval is also susceptible to methodological differences, population characteristics and geographical differences, the reference interval provided by the reagent manufacturer has limitations, so a laboratory self‐built reference interval is particularly important.^12^, ^13^ Unfortunately, so far there have been a few reports about the establishment of SAA reference intervals in humans.^14^, ^15^ Hence, the present study was conducted through recruitment, sampling, and measurement. Meanwhile, we collected data of healthy subjects, conducted preliminary analyses, and established the reference interval of SAA by using latex‐enhanced immunoturbidimetry for healthy adults in China. These reference values can be used for the clinical application of SAA‐related diseases.

## 2 MATERIAL AND METHODS

### 2.1 Study subjects

**Figure 1:**
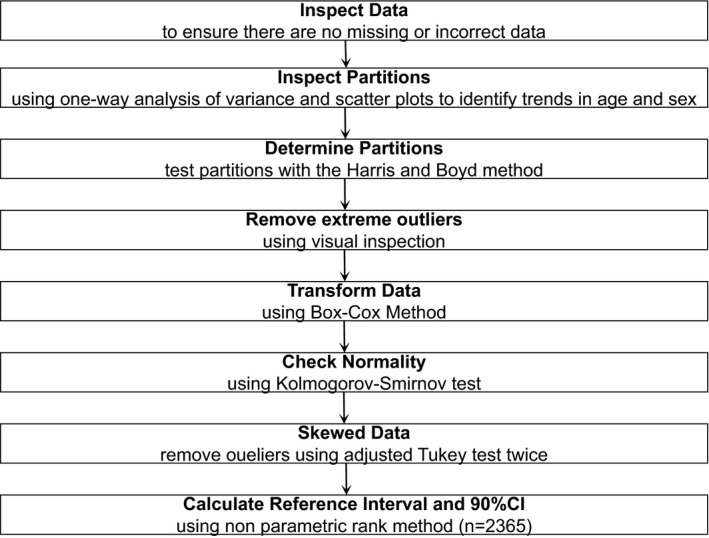
Establishing a reference interval of SAA on the bias of CLSI CA28‐A3

According to the principle of complete randomness, 2600 subjects who completed a physical examination at the Physical Examination Center of the Second People's Hospital of Lianyungang from January 2019 to February 2019 were selected as participants in this study. Based on inclusion and exclusion criteria, a total of 2365 reference individuals (1152 males and 1213 females; 20‐79 years of age) conformed to the requirements of this study. In accordance with the guidelines of CLSI C28‐A3,^16^ all subjects were divided into two or six groups by sex (male and female) or age (20‐29 years of age; 30‐39 years of age; 40‐49 years of age; 50‐59 years of age; 60‐69 years of age; and 70‐79 years of age). The detailed screening procedures of the study participants are shown in Figure 1.

Participants in this study met the following inclusion criteria: (a) 20‐79 years of age; (b) blood pressure ≤ 139/89 mm Hg; (c) body mass index of 18.5‐28.0 kg/m^2^; (d) routine biochemical analytes, such as C‐reactive protein (CRP), total bilirubin (TBIL), total protein (TP), albumin (ALB), alanine aminotransferase (ALT), aspartate aminotransferase (AST), gamma‐glutamyltransferase (GGT), urea, creatinine (Cr), glucose (GLU), total cholesterol (TC), triglyceride (TG), high‐density lipoprotein cholesterol (HDL‐C), and low‐density lipoprotein cholesterol (LDL‐C), were within the normal reference intervals (Table 1), and (e) the urine routine analytes were normal. Participants were excluded if they had the following: (a) acute trauma, acute or chronic inflammation, or infectious diseases; (b) primary or secondary liver disease or kidney disease; (c) various autoimmune diseases and endocrine disorders; (d) recent medication, surgery, and other treatments; and (e) recent irregular work schedules, insufficient sleep, or excessive alcohol consumption. This study was approved by the Medical Ethics Committee of the Second People's Hospital of Lianyungang (2017‐013‐01), and informed consents were signed by all participants.

**Table 1:** Clinical characteristics of the subjects

Analytes	Test results ($\bar{x}\;\pm \;s$)	Reference interval	Units of measurement
CRP	5.6 ± 2.1	0.0‐10.0	mg/L
TBIL	12.2 ± 2.3	3.4‐17.1	μmol/L
TP	74.4 ± 3.6	65.0‐85.0	g/L
ALB	47.1 ± 2.8	40‐55	g/L
ALT	26 ± 6	9‐50 (male) 7‐40 (female)	U/L
AST	25 ± 6	15‐40 (male) 13‐35 (female)	U/L
GGT	28 ± 7	10‐60 (male) 7‐45 (female)	U/L
Urea	5.2 ± 1.9	3.1‐8.0 (male, ages 20‐59) 3.6‐9.5 (male, ages 60‐79) 2.6‐7.5 (female, ages 20‐59) 3.1‐8.8 (female, ages 60‐79)	mmol/L
Cr	55 ± 9	57‐97 (male, ages 20‐59) 57‐111 (male, ages 60‐79) 41‐73 (female, ages 20‐59) 41‐81 (female, ages 60‐79)	μmol/L
GLU	4.89 ± 0.45	3.60‐6.10	mmol/L
TC	4.65 ± 0.43	<5.17	mmol/L
TG	1.12 ± 0.34	<1.70	mmol/L
HDL‐C	1.23 ± 0.18	0.91‐1.66 (male) 0.91‐1.74 (female)	mmol/L
LDL‐C	2.75 ± 0.42	<3.37	mmol/L

Note

Abbreviations: ALB, albumin; ALT, alanine aminotransferase; AST, aspartate aminotransferase; Cr, creatinine; CRP, C‐reactive protein; GGT, gamma‐glutamyltransferase; GLU, glucose; HDL‐C, high‐density lipoprotein cholesterol; LDL‐C, low‐density lipoprotein cholesterol; TBIL, total bilirubin; TC, total cholesterol; TG, triglyceride; TP, total protein.

### 2.2 Specimen collection

A peripheral venous blood sample (5 mL) was drawn in the morning. The specimens were centrifuged at 1200 *g* for 10 minutes, and the detection of all analytes was completed within 4 hours. At the same time, 10 mL of fresh morning urine was collected, and the measurement of urine was completed within 2 hours.

### 2.3 Instruments and reagents

According to the reagent instructions, the levels of TBIL, TP, ALB, ALT, AST, GGT, urea, Cr, GLU, TC, TG, HDL‐C, and LDL‐C were detected by an AU5821 automatic biochemical analyzer (Beckman Coulter) with corresponding reagents (TBIL kit lot: AUZ6008; TP kit lot: AUZ5261; ALB kit lot: AUZ5259; ALT kit lot: AUZ5486; AST kit lot: AUZ5921; GGT kit lot: AUZ5930; urea kit lot: AUZ5951; Cr kit lot: 2453; GLU kit lot: AUZ5183; TC kit lot: AUZ5424; TG kit lot: AUZ5480; HDL‐C kit lot: AUZ5523; and LDL‐C kit lot: AUZ5716; Beckman Coulter). Meanwhile, the level of CRP was detected by IMMAGE 800 specific protein analysis system (Beckman Coulter) with its corresponding reagents (CRP kit lot: M810256). The level of SAA was also detected using an AU5821 automatic biochemical analyzer (Beckman Coulter., Ltd.) with matching reagents (SAA kit lot: 20 181 026; Zhejiang Zhuoyun Biotechnology). Urinalysis was conducted using an UN2000 automatic urine analyzer (Sysmex) with matching reagents. To minimize analytical errors, the same batches of reagents were used throughout the whole process of detection. All instruments and equipment were maintained and calibrated according to the manufacturer's instructions, and the internal quality control was in control and external quality assessment was qualified prior to the detection of samples.

### 2.4 Statistical analyses

#### 2.4.1 Outlier test

According to the Dixon method recommended by the guidelines of CLSI C28‐A3,^16^ the detection results were sorted by size (from small to large) to calculate the range, R. The distribution was then used to calculate the difference (D) between the maximum value or the minimum value and its adjacent value. If the D/R was ≥ 1/3, then the maximum or minimum value was eliminated as an outlier; the remaining data were used in the above steps until all outliers were eliminated.

#### 2.4.2 Identification of biological reference interval groups

According to the recommended methods of CLSI C28‐A3 and WS/T 402‐2012 (“Establishment of reference interval for clinical laboratory test items”),^16^, ^17^ the *Z* test was used to determine whether the data from two comparison groups could be combined. The formula for the *Z* test is as follows:

$$Z\;=\;\frac{{\bar{x}}_{1}-{\bar{x}}_{2}}{\sqrt{\frac{S_{1}^{2}}{n_{1}}\;+\;\frac{S_{2}^{2}}{n_{2}}}},\;Z^{*}\;=\;3\sqrt{\frac{n_{1}\;+\;n_{2}}{240}}$$

where and ${\bar{x}}_{1}$ and ${\bar{x}}_{2}$ represent the mean values from the two groups, *s*_1_ and *s*_2_ represent the standard deviations from the two groups, and *n_1_* and *n_2_* represent the sample sizes of the two groups. If *Z* is > Z*, then the difference between the reference intervals is statistically significant (*P *< .05), and the reference intervals for the two groups should remain separate. In contrast, if *Z* is <Z*, then the difference between the two reference intervals is not statistically significant (*P*> .05), and the intervals can be merged into one reference interval.

### 2.5 Statistical analysis

The data were analyzed using SPSS Statistics, version 20 (IBM). The Kolmogorov‐Smirnov test was used to analyze the normality of the data. Nonparametric data were expressed using the median and interquartile range (IQR), and the Kruskal‐Wallis *H* test was used to compare across multiple groups. A nonparametric (ranked) method was used to establish reference intervals according to CLSI C28‐A3. The difference was statistically significant at *P *< .05.

## 3 RESULTS

**Figure 2:**
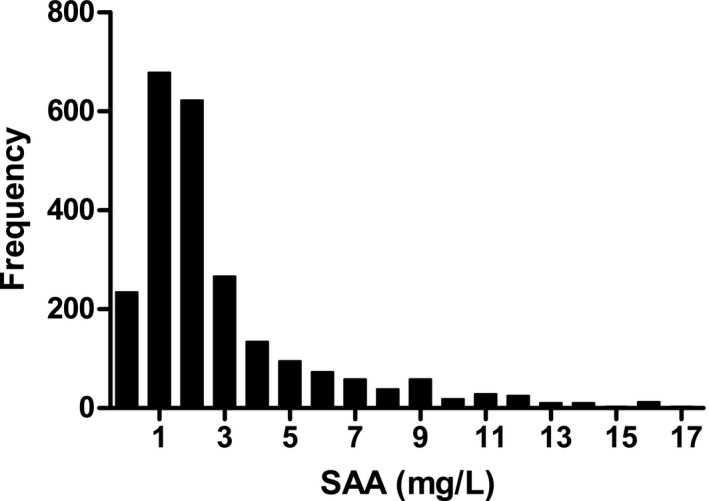
Distribution histogram of serum amyloid A (SAA) levels

A total of 2365 subjects were ultimately retained from the inclusion and exclusion criteria in this study, which included 1152 males and 1213 females. The clinical characteristics of the subjects were listed in Table 1. The Kolmogorov‐Smirnov test showed a skewed distribution for the concentrations of SAA (*P *< .05; Figure 2). The distribution of SAA levels was not significantly different between males and females, which indicated that the concentrations of SAA were not associated with sex (*Z *= 0.30, Z* = 9.42, *P*> .05; Table 2). No significant age‐related differences in the levels of SAA were found, which showed that the data of SAA were not correlated with age (*P*> .05; Table 3).

**Table 2:** Distribution of serum amyloid A (SAA) by sex

Sex	n	Median (IQR)	Upper limit	*Z* test
Male	1152	1.8 (1.0‐3.2)	10.4 (90% CI: 9.2‐13.6)	*Z *< *Z* ^*^ *P *> .05
Female	1213	1.9 (1.1‐3.5)	11.4 (90% CI: 9.3‐13.6)	
Total	2365	1.9 (1.0‐3.4)	11.0 (90% CI: 9.3‐12.3)	

Note

The unit of SAA is mg/L.

Abbreviations: CI, confidence interval; IQR, interquartile range.

**Table 3:** Distribution of serum amyloid A (SAA) by age

Age group	n	Median (IQR)	Upper limit	*H*‐value	*P*‐value
Aged 20‐29	365	1.8 (0.9‐3.2)	9.5 (90% CI: 6.7‐15.3)	8.04	.15
Aged 30‐39	353	1.9 (1.0‐3.5)	10.9 (90% CI: 7.4‐16.2)		
Aged 40‐49	428	1.8 (0.9‐3.2)	11.00 (90% CI: 7.9‐15.1)		
Aged 50‐59	453	2.1 (1.1‐4.2)	12.0 (90% CI: 8.9‐16.5)		
Aged 60‐69	395	1.9 (1.2‐3.2)	10.9 (90% CI: 7.5‐16.6)		
Aged 70‐79	371	1.8 (1.1‐3.4)	12.2 (90% CI: 8.0‐15.9)		

Note

The H‐value is the approximate chi‐square value.

The unit of SAA is mg/L.

Abbreviations: CI, confidence interval; IQR, interquartile range.

In accordance with nonparametric methods recommended by CLSI C28‐A3 and WS/T 402‐2012, and combined with the clinical value of SAA, the upper reference limit was very important, which was set at the 95th percentile. The population reference interval of the SAA was established as an upper limit of 11.0 mg/L (90% confidence interval: 9.3‐12.3 mg/L) by using the method of latex immunoturbidimetry in this study.

## 4 DISCUSSION

Serum amyloid A is an important acute‐phase reactive protein mainly produced by liver, which can eliminate pathogens in the body and facilitate disease recovery.^18^, ^19^ And thus, it is a very sensitive marker reflecting an acute inflammatory state.^20^ This study combined the regional characteristics of population distribution and eating habits in coastal cities, established strict normative inclusion and exclusion criteria for reference individuals, and finally confirmed 2365 healthy people with physical examinations as the research cohort. Our research showed that the distribution of SAA in healthy adult females was slightly higher than that in healthy adult males, but there was no significant difference (*P*> .05). This finding is consistent with a previous study by Wu et al,^21^ which reported no difference in SAA level was found between genders. Besides, our research also conducted statistical analysis based on age. The results showed that there was no significant difference in the level of serum SAA among healthy adults in terms of age (*P*> .05).

In view of the lower limit of the reference interval of SAA does not have clinical diagnostic significance, this preliminary study established the SAA population reference interval for the upper limit 11.0 mg/L (90% confidence interval: 9.3‐12.3 mg/L), which is basically consistent with the reference interval stated by Zhuoyun Biotechnology. The upper limit of the reference interval provided by the reagent manufacturer of the Zhuoyun SAA kit is 10.0 mg/L. In a similar study, Wu et al^21^ conducted a serum SAA reference interval research among healthy subjects (n = 201) ranging from 30 to 90 years and established the SAA reference interval for the upper cutoff of 6.75 mg/L (P_95_). This difference may be due to different methodologies. In this study, the method of latex‐enhanced immunoturbidimetry was used to detect SAA, while Wu et al^21^ used enzyme‐linked immunosorbent assay (ELISA) to detect SAA. Additionally, Fellahi et al^22^ conducted a study over a span of 5 years (2014‐2018), based on data from the population reference interval (SAA ≤ 6.4 mg/L) provided by the reagent manufacturer when combined with C‐reactive protein (CRP). The results of the paired study showed that when CRP was < 5 mg/L, SAA ≤ 6.4 mg/L and SAA ≤ 10.0 mg/L accounted for 84.6% and 92.8% of the total data, respectively. When CRP was < 3 mg/L, the data of SAA ≤ 6.4 mg/L and SAA ≤ 10.0 mg/L accounted for 89.6% and 95.6% of the total results, respectively. The results of Fellahi et al^22^ further showed that the distribution of SAA results did not differ statistically among children, adults, and all populations (*P*> .05). There were some differences between the population reference interval of SAA reported by Fellahi et al and our study. The population reference interval of SAA provided by Fellahi et al^22^ was significantly lower than the population reference interval established by this study. On the contrary, Ichihara et al^15^ conducted a survey of SAA reference interval showed that the upper limit of SAA reference interval was higher than that of this study for either Asian or Chinese populations. The reasons for these differences may be as follows: (a) different detection methods; SAA was mainly detected by immunoturbidimetry, enzyme‐linked immunosorbent assay, or microsphere capture enzyme immunoassay 23, 24, 25; (b) there were some differences in population characteristics and regional characteristics, such as race, environment, and living habits.^26^, ^27^ All of the above parameters may have resulted in different results. The results of this study suggested that every laboratory should establish a SAA population reference interval that accounts for the regional population, factors in the regional environment, and detection system characteristics.

However, there were some limitations in this study. First, this study was a single‐center study and all subjects were from coastal areas, so the population distribution was relatively single. Second, the age of the subjects in this study was not fully covered, and there were no data on the part of the subjects younger than 20 years old. Thus, in future studies, we plan to adopt multicenter research and cover all age groups of the subjects to ensure that the research results are more representative.

In conclusion, SAA is a sensitive acute‐phase reactive protein, which is closely related to the occurrence and progression of various diseases. Therefore, the preliminary establishment of a population reference interval for SAA can fully exert its potential clinical value.

## AUTHORS’ CONTRIBUTIONS

QL and YL researched literature and conceived the experiments. TDX, LY, JS, and WL were involved in protocol development, gaining ethical approval, patient recruitment, and data analysis. QL, YL, and FMY wrote the first draft of the manuscript. All authors reviewed and edited the manuscript and approved the final version of the manuscript.
